# Characterization of Human Breast Milk-Derived *Limosilactobacillus reuteri* MBHC 10138 with Respect to Purine Degradation, Anti-Biofilm, and Anti-Lipid Accumulation Activities

**DOI:** 10.3390/antibiotics13100964

**Published:** 2024-10-12

**Authors:** Jinhua Cheng, Joo-Hyung Cho, Joo-Won Suh

**Affiliations:** Microbio Healthcare, Co., Ltd., Yongin 17058, Republic of Korea; jhcheng316@mju.ac.kr (J.C.); chovincent@mju.ac.kr (J.-H.C.)

**Keywords:** *Limosilactobacillus reuteri*, breast milk, probiotics, anti-biofilm, purine degradation, anti-lipid accumulation

## Abstract

**Background:** Human breast milk is a valuable source of potential probiotic candidates. The bacteria isolated from human breast milk play an important role in the development of the infant gut microbiota, exhibiting diverse biological functions. **Methods:** In this study, *Limosilactobacillus reuteri* MBHC 10138 isolated from breast milk was characterized in terms of its probiotic safety characteristics and potential efficacy in hyperuricemia, obesity, lipid liver, and dental caries, conditions which Korean consumers seek to manage using probiotics. **Results:** Strain MBHC 10138 demonstrated a lack of D-lactate and biogenic amine production as well as a lack of bile salt deconjugation and hemolytic activity. It also exhibited susceptibility to common antibiotics, tolerance to simulated oral–gastric–intestinal conditions, and superior biological activity compared to three *L. reuteri* reference strains, including KACC 11452 and MJ-1, isolated from feces, and a commercial strain isolated from human breast milk. Notably, *L. reuteri* MBHC 10138 showed high capabilities in assimilating guanosine (69.48%), inosine (81.92%), and adenosine (95.8%), strongly inhibited 92.74% of biofilm formation by *Streptococcus mutans*, and reduced lipid accumulation by 32% in HepG2 cells. **Conclusions:** These findings suggest that strain MBHC 10138, isolated from human breast milk, has potential to be developed as a probiotic for managing hyperuricemia, obesity, and dental caries after appropriate in vivo studies.

## 1. Introduction

Human breast milk is a rich source of beneficial bacteria, including various strains of lactic acid species such as *Lactobacillus*, *Bifidobacterium*, and *Streptococcus* [1]. These probiotic bacteria are naturally transferred from the mother to the infant during breastfeeding, contributing to the establishment of the latter’s gut microbiota. Furthermore, they play a significant role in immune system maturation and exert a multifaceted beneficial impact on metabolic disorders through gut microbiota modulation, enhancement of its barrier function, reduction in inflammation, and direct metabolic effects [2,3].

Among these bacteria, *Lactobacillus* strains isolated from human breast milk have garnered significant interest due to their potential health benefits as probiotics. They contribute to the establishment and maintenance of a healthy gut microbiota, enhance immune function, and protect against various health conditions [4,5]. For instance, *Lactobacillus reuteri* DSM17938, which was originally isolated from the breast milk of a Peruvian mother, plays a pivotal role in improving gut health by promoting the growth of beneficial bacteria and inhibiting pathogenic species, leading to better digestion and nutrient absorption. This strain also significantly reduces crying time in colicky infants, enhancing their overall quality of life [6]. *Lactobacillus fermentum* CECT5716, a strain isolated from human milk, enhances the maturation of the infant’s immune system, providing robust protection against common infections and reducing the frequency and severity of respiratory and gastrointestinal ailments [7]. Recent studies have shown that certain probiotics derived from human breast milk exhibit superior probiotic properties compared to those sourced from other environments, potentially due to their adaptation to the human gut [1,7].

Previous studies have reported that infants who were exclusively breastfed were less likely to be overweight at 1 year of age than those who received formula early in life. Differences in the intestinal microbiome characteristics between breastfed and formula-fed infants at 3 months of age suggest that the gut microbiota of breastfed infants plays a crucial role in protecting against obesity [1,8]. Thus, the role of breast milk-derived probiotics in obesity is worthy of study.

Like obesity, hyperuricemia, a status of elevated uric acid in the serum, is associated with many metabolic disorders [9] and reportedly associated with an excessive intake of high-purine food; eliminating purine nucleosides may thus relieve people from this condition [10]. Many probiotics, particularly those from the genera *Lactobacillus* and *Bifidobacterium*, have been shown to possess nucleoside-degrading capabilities thanks to their production of various enzymes like nucleoside phosphorylases, nucleoside hydrolases, and nucleotidases [11].

Not only in the gut, probiotics also play an important role in the oral cavity. *Lactobacillus rhamnosus* GG (LGG), a well-researched probiotic strain, is attracting growing interest for its potential role in oral health, including in dental caries’ prevention [12,13]. LGG was reported to inhibit the bacteria responsible for dental caries, such as *Streptococcus mutans*, which is one of the main pathogens in tooth decay, by competing with harmful bacteria for adhesion sites on the teeth and gums, modulating the oral microbiome, or producing antimicrobial substances to inhibit the growth of cariogenic strains [14].

Probiotics are live microorganisms that confer health benefits when administered in adequate amounts. Ensuring their safety is paramount for clinical and consumer use. According to the Korean Food and Drug Administration (Korean FDA) guideline, probiotics should neither show hemolytic activity and cell toxicity nor harbor transferable antibiotic resistance genes. They should also have low D-lactate production and bile salt deconjugation activities. Finally, they should also survive transit through the gastrointestinal tract, resisting stomach acid and bile.

In this study, human breast milk-isolated *Lactobacillus reuteri* MBHC 10138 was studied for its safety and probiotic characteristics, as required by the Korean FDA, to determine its biogenic amine, hemolytic activity, bile salt deconjugation, D-lactate production, antibiotic susceptibility, and viability in simulated oral–gastric–intestinal transit conditions. *Lacticaseibacillus rhamnosus* GG (LGG), a widely studied probiotic, was used as a reference strain for determining the safety and probiotic characteristics.

Furthermore, to meet the need for using probiotics to manage hyperuricemia, oral health, and fatty liver in the Korean population, MBHC 10138’s purine degradation, anti-biofilm activity against *S. mutans,* and inhibitory effect on lipid accumulation in HepG2 cells were investigated. For comparison, three reference *L. reuteri* strains with different origins were tested at the same time. The reference strain MJ-1, isolated from a baby’s fecal sample, was provided by Microbiohealthcare Co., Ltd., from a bank of microbial collections (Yongin, Republic of Korea). The commercial strain (*L. reuteri* DSM17538), originally isolated from breast milk, was obtained from a commercial probiotic product (Biogaia Protectis Baby Drop) and identified via the 16S rDNA sequence. Finally, the strain *L. reuteri* KACC 11452 (fecal origin) was purchased from the Korean Agricultural Culture Collection (KACC). All the strains are close in the phylogenetic tree.

## 2. Results

### 2.1. L. reuteri MBHC 10138 Identification

The 16S rDNA sequence amplified using primers 27F and 1492R was blasted on EZbiocloud.net, an integrated platform representing the taxonomic hierarchy of bacteria and archaea through quality-controlled 16S rRNA genes [15]. Sequences showing an over 97% similarly to MBHC 10138 were used to construct the phylogenetic tree. For comparison, the sequences of the MJ-1, commercial, and KACC 11452 strains were also included.

**Figure 1:**
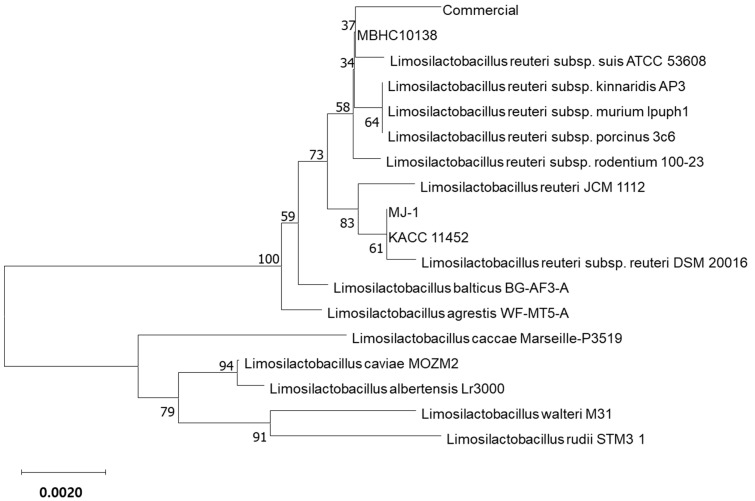
A phylogenic tree based on the 16S-rRNA gene sequence, showing *Limosilactobacillus reuteri* MBHC 10138’s relationship with the closely related *Limosilactobacillus* species. The sequences of related species that were over 97% similar to MBHC 10138 were downloaded from the Ezbiocloud database. The tree was generated by the Mega program (Version 11) using the neighbor-joining method. The numbers at the nodes indicate the percentage levels of bootstrap support based on a neighbor-joining analysis of 1000 replicas. The scale bar denotes 0.002 substitutions per nucleotide position.

A phylogenetic analysis identified MBHC 10138 as *Limosilactobacillus reuteri*, revealing a close relationship with the newly recognized subspecies *L. reuteri* subsp. *suis* ATCC 53608 (Figure 1). The results indicated that the four strains clustered into two distinct groups: *L. reuteri* commercial strain and MBHC 10138 exhibited a close association with *Limosilactobacillus reuteri* subsp. *suis* ATCC 53608, while strains MJ-1 and KACC 11452 were more closely related to *Limosilactobacillus reuteri* subsp. *reuteri* DSM 20016.

### 2.2. Safety Assessment and Probiotic Characterization of L. reuteri MBHC 10138

#### 2.2.1. *L. reuteri* MBHC 10138 Biogenic Amine Production

Strain MBHC 10138 was tested for biogenic amine production on the decarboxylase medium supplemented with L-arginine, L-histidine, L-lysine, L-ornithine, L-phenylalanine, L-tyrosine, and L-tryptophan. No significant production of biogenic amine was observed in these amino acids for the strain MBHC 10138 (Table 1, Figure S1).

#### 2.2.2. Hemolytic Activity

Hemolytic activity refers to the ability of an organism to lyse red blood cells, which can be divided into α-hemolysis (the green zones around colonies), β-hemolysis (clear zones around the colony), and γ-hemolysis (no zone). Some strains of Lactobacillus have been found to exhibit weak or α-hemolytic activity [16], although probiotics should ideally show none. In this study, *E. coli* O138, a positive control, showed a clear zone on the blood agar, while strain MBHC 10138 showed none, indicating no hemolytic activity (Table 1, Figure S2).

#### 2.2.3. Bile Salt Deconjugation

Bile salt deconjugation often releases free bile acids, which can precipitate with calcium chloride and ferric chloride, forming a visible zone around the colonies. The presence of clear zones or precipitation around probiotic colonies therefore indicates bile salt deconjugation activity. In our study, there was no obvious clear zone or precipitation around the colonies compared to strain LGG, suggesting that MBHC 10138 lacks bile salt deconjugation activity (Table 1).

#### 2.2.4. D-Lactate Production

D-lactate is a stereoisomer of lactic acid that can accumulate in the bloodstream if produced in excess. *L. reuteri* MBHC 10138 produced 18.39 g/L of D-lactate and 95.77 g/L of L-lactate, while LGG supplied 23.11 g/L and 143.77 g/L (Table 1).

#### 2.2.5. Antibiotic Susceptibility

*L. reuteri* MBHC 10138 showed antibiotic susceptibility to chloramphenicol, clindamycin, erythromycin, and tetracycline, with MIC values of 4, 0.25, 1, and 16 μg/mL, meeting the EFSA cut-off. However, the strain was resistant to ampicillin, gentamicin, kanamycin, and streptomycin (Table 1).

#### 2.2.6. Tolerance to Oral–Gastric–Intestinal Transit Stress

MBHC 10138 survival under oral, gastric, and intestinal stresses was tested with an oral–gastric–intestinal (OGI) transit assay. The cell viability of MBHC 10138 and LGG was maintained under oral stress but was affected by the other two stresses. The log CFU of MBHC 10138 decreased to 9.11 ± 0.04 and 9.04 ± 0.030 after being treated with gastric solutions with pH 3.0 and 2.0, respectively. After being treated with an intestinal solution, the log CFU further decreased to 8.58 ± 0.050. The log CFU of LGG also decreased from 9.21 ± 0.023 to 9.21 ± 0.006, 9.17 ± 0.103, 9.01 ± 0.035, and 7.98 ± 0.045 after being treated with oral, gastric (pH 3.0 and pH 2.0), and intestinal solutions, respectively (Table 2).

### 2.3. Nucleoside Degradation of L. reuteri Strains

**Figure 2:**
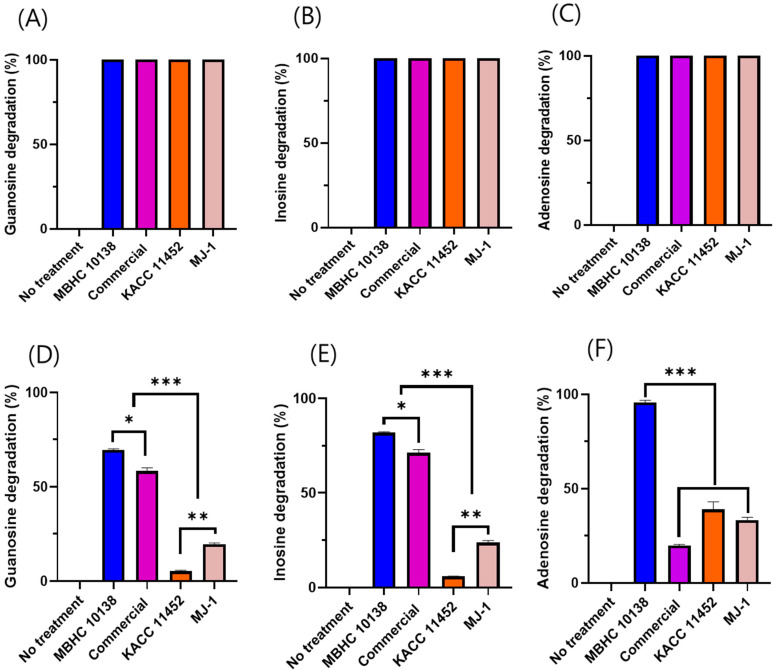
*Limosilactobacillus reuteri* MBHC 10138, commercial, KACC 11452, and MJ-1 strains’ guanosine (**A**,**D**), inosine (**B**,**E**), and adenosine (**C**,**F**) degradation ability. The cell density is 10^9^ CFU/mL (**A**–**C**), 10^8^ CFU/mL (**D**,**E**), and 10^7^ CFU/mL (**F**), for 30 min (**A**–**C**) or 15 min (**D**–**F**) of incubation. The results are presented as the mean ± standard deviation of triplicate independent experiments. * *p* < 0.05, ** *p* < 0.01, and *** *p* < 0.001.

Nucleoside degradation by the *L. reuteri* MBHC 10138, commercial, KACC 11452, and MJ-1 strains was initially tested at a 10^9^ cfu/mL cell density for 30 min of incubation. All strains demonstrated 100% nucleoside degradation (Figure 2A–C). To further differentiate the degradation capabilities of the various strains, the cell density was decreased to 10^8^ cfu/mL and 10^7^ cfu/mL for the degradation of guanosine/inosine and adenosine, respectively, for 15 min of incubation.

Under these modified conditions, *L. reuteri* MBHC 10138 exhibited superior nucleoside degradation abilities, achieving rates of 69.48%, 81.92%, and 95.8% for guanosine, inosine, and adenosine, respectively, within 15 min. In contrast, the commercial strain’s degradation rates for guanosine, inosine, and adenosine were 58.36%, 71.18%, and 19.8%, respectively. Meanwhile, strains KACC 11452 and MJ-1 showed relatively low degradation rates for guanosine and inosine; specifically, the former achieved 5.31%, 5.87%, and 39% degradation for guanosine, inosine, and adenosine, while the latter recorded rates of 19.43%, 23.75%, and 33% (Figure 2D–F). These results indicate that strain MBHC 10138 has a notably higher capability for degrading purines, particularly adenosine, compared to the other strains.

### 2.4. Streptococcus mutans KCTC3065 Biofilm Inhibition

**Figure 3:**
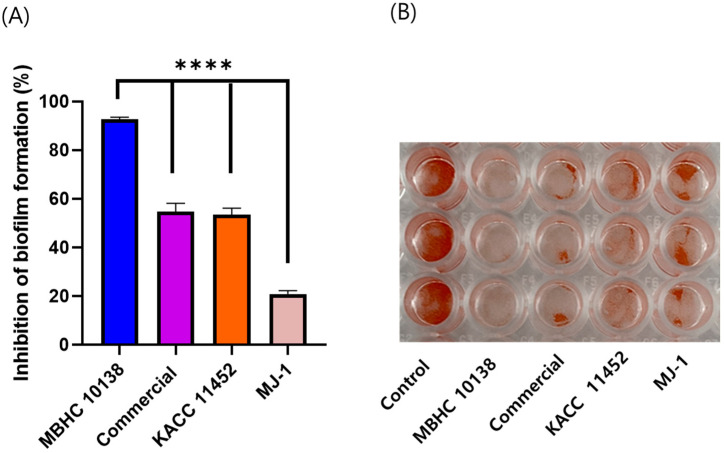
*Streptococcus mutans* KCTC 3065 biofilm formation was inhibited by treatment with *Limosilactobacillus reuteri* MBHC 10138, commercial, KACC 11452, and MJ-1 strains, at 10^8^ cfu/mL concentrations. Biofilm inhibition by *L. ruteri* was quantified via comparison with the untreated control (**A**), and the safarin-dyed biofilm was shown on the plates (**B**). The results are presented as the mean ± standard deviation of triplicate independent experiments. **** *p* < 0.0001.

*Streptococcus mutans* KCTC 3065 biofilm formation was inhibited when co-cultured with *L. reuteri* strains, with MBHC 10138 showing the strongest inhibition rate of 92.74%. The inhibition rates of the commercial, MJ-1, and KACC 11452 strains were 54.66%, 20.84%, and 53.56%, respectively (Figure 3).

### 2.5. Effect of L. reuteri Strains on Lipid Accumulation in HepG2 Cells

**Figure 4:**
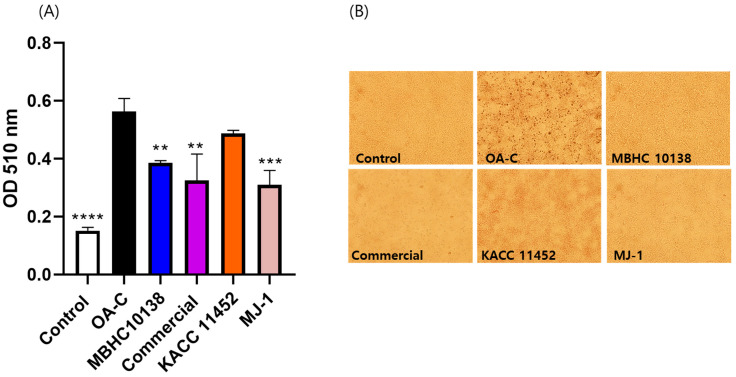
Lipid accumulation inhibition by *Limosilactobacillus reuteri* MBHC 10138, commercial, KACC 11452, and MJ-1 treatment in HepG2 cells at a cell density of 10^9^ cfu/mL; those stained with Oil Red O were extracted with isopropanol and quantified at OD 510 (**A**). A typical image of stained HepG2 cells under an optical microscope at 100× magnification (**B**). The results are presented as the mean ± standard deviation of triplicate independent experiments. ** *p* < 0.01, *** *p* < 0.001 and **** *p* < 0.0001.

Lipid accumulation was assessed in HepG2 cells, whereby those that had been treated with a fatty solution presented a 303% increase. Treatment with the MBHC 10138, commercial, KACC 11452, and MJ-1 strains significantly reduced lipid accumulation by 29%, 32%, 12%, and 33%, respectively (Figure 4), indicating *L. reuteri*’s potential anti-obesity activity, despite this being strain-specific.

## 3. Discussion

In this study, the safety and probiotic characteristics of human breast milk-isolated *L. reuteri* MBHC 10138 were evaluated. Furthermore, its nucleoside degradation, anti-biofilm activity, and lipid accumulation inhibition in HepG2 cell were compared with those of reference *L. reuteri* strains.

According to the phylogenetic analysis, MBHC 10138 and the commercial strain are closely related, while KACC 11452 and MJ-1 are closely related. This grouping was also evident in the phenotypic characteristics, with clear variations in colony morphology: MBHC 10138 and the commercial strain displayed round, plump, shiny milky white colonies, whereas those of MJ-1 and KACC 11452 were faint yellow, and translucent (Figure S3). These findings underscore the morphological and genetic diversity within *Limosilactobacillus reuteri*.

Interestingly, the above grouping was also reflected in the isolation origins: the MBHC 10138 and commercial strains were isolated from breast milk, while KACC 11452 and MJ-1 originated from feces. Recently, many *L. reuteri* strains have been reclassified into new species or subspecies based on whole-genome DNA–DNA hybridization values [17]. Further investigation of additional strains is needed to determine whether specific subspecies are consistently isolated from distinct sources.

*L. reuteri* strains are frequently isolated from human milk. For instance, it has been reported that 50% of mothers from rural areas in Japan and Sweden were *L. reuteri*-positive, with 15% on average having detectable *L. reuteri* in their milk [18]. Due to the frequent occurrence of these bacteria in breast milk, it is meaningful to explore their biological function.

Safety is the primary requirement for a probiotic strain. In this study, a safety assessment was carried out by determining the strains’ hemolytic and bile salt deconjugation activity, biogenic amine and D-lactate production, and antibiotic susceptibility.

Biogenic amines (BAs) are organic nitrogen compounds produced by various microorganisms, including probiotics, mainly through amino acid decarboxylation. Some biogenic amines, like spermidine and spermine, are essential for cell growth and differentiation. However, high levels of other amines (e.g., histamine and tyramine) can cause adverse effects like headaches, hypertension, and allergic reactions [19,20,21]. MBHC 10138 showed no biogenic amine production and can, therefore, be used as a probiotic (Table 1).

Bile salt emulsifies fat and promotes the action of lipase, helping fatty acid decomposition. Bile salt hydrolase in probiotics protects lactic acid bacteria, increasing their intestinal colonization by deconjugating bile salt. However, this removes their emulsifying function, making it difficult to decompose fatty acids. Furthermore, excessive deconjugation can lead to the formation of secondary bile acids, whose high concentration has been linked to colorectal cancer and other gastrointestinal disorders [22]. MBHC 10138 did not show more bile salt assimilation than LGG, a well-known probiotic strain, indicating its safety in bile salt deconjugation (Table 1).

Some probiotics, particularly *Lactobacillus strains*, produce D-lactate as a fermentation byproduct. In individuals with an impaired lactate metabolism, such as those with short bowel syndrome or immature metabolic pathways (e.g., infants), excessive D-lactate concentrations can lead to D-lactic acidosis. Thus, a high level of D-lactate production is not desirable for probiotics. In this study, *L. reuteri* MBHC 10138 produced less D-lactate than LGG, showing its safety (Table 1).

Probiotics’ antibiotic resistance genes pose the risk of being transferred to pathogenic bacteria, potentially leading to the development and spread of antibiotic-resistant infections. On the other hand, if a probiotic shows resistance, it can be administered in conjunction with antibiotic treatments, especially to restore gut flora. MBHC 10138 showed resistance to aminoglycoside antibiotics, which is generally very high in probiotics [23]. In future works, it is thus necessary to determine whether this resistance is intrinsic, e.g., due to chromosomal mutations, or acquired via horizontal gene transfer.

Probiotics can produce a variety of nucleoside-degrading enzymes, like nucleoside phosphorylases and hydrolases and nucleotidases. These enzymes can convert nucleosides into purine bases, which have lower water solubility and can thus be excreted from the body via feces [11].

In a previous study, *Lactobacillus reuteri* TSR332 and *Lactobacillus fermentum* TSF331 showed guanosine and inosine assimilation abilities. In particular, the former metabolized 90.05% of inosine and 78.02% of guanosine over 30 min [24]. In our study, MBHC 10138 showed higher nucleoside assimilation than *L. reuteri* TSR332, removing 100% of guanosine and inosine in 30 min (Figure 2A–C). This indicated that this strain could be developed as a probiotic for ameliorating hyperuricemia.

Among the microbes living in the oral cavity, *S. mutans* is considered the main pathogen in the initiation and development of dental caries. The biofilm formed by *S. mutans* provides a unique microenvironment for bacterial growth, metabolism, and survival, enabling *S. mutans* to be more resistant to harsh oral conditions. Thus, targeting this biofilm may be an alternative approach to preventing dental caries without worrying about antibiotic resistance [25].

Probiotics inhibit bacterial biofilm formation through several mechanisms, including the production of antimicrobial substances [26], competition for adhesion sites [27], modulation of the host immune response [28], and interference in quorum sensing. In this study, *L. reuteri* MBHC 10138 demonstrated a significantly stronger inhibitory activity against *Streptococcus mutans* biofilm formation compared to the other strains tested. These results showed MBHC 10138’s potential to be used as an oral probiotic when administered as a living organism. To ensure viability, future studies on formulations such as powders or particles with a freeze-drying protectant are necessary. In addition, MBHC 10138’s marked biofilm inhibition suggests a unique or enhanced mechanism of action which warrants further investigation in future studies to fully understand its underlying processes and potential applications.

Many *Lactobacillus* strains have been reported to inhibit lipid accumulation in HepG2 cells, a commonly used model for studying liver metabolism and obesity. Previous studies have suggested that *L. reuteri* can modulate the lipid metabolism and reduce lipid synthesis. For instance, *L. reuteri* NCIMB 30242 was found to significantly decrease cholesterol accumulation in HepG2 cells by altering the expression of genes involved in lipid metabolism, such as HMG-CoA reductase and AMPK [29]. Additionally, another study suggested that *L. reuteri* TSR332 might reduce lipid accumulation by lowering inflammation and oxidative stress in liver cells, providing a multifaceted approach to managing lipid metabolism, contributing to overall liver health [30]. These activities are strain-specific.

Probiotic metabolites have also been reported to inhibit lipid accumulation in HepG2 cells. It is well established that probiotics exert their effect through the production of metabolites such as short-chain fatty acids (SCFAs), which are, in turn, known to interact with free fatty acid receptors, stimulating the secretion of glucagon-like peptide-1, an incretin mediating the host’s postprandial effect to regulate energy homeostasis. Additionally, previous research has shown that lactate plays an important role in the regulation of energy metabolism via AMPK activation [31]. Another study reported that extracellular vesicles from probiotics can contribute to their beneficial effects [32]. In this study, the differences in the activity of distinct strains might have been due to different small molecular metabolites or cell-derived macromolecules.

Many probiotics inhibiting lipid accumulation in HepG2 cells also exerted anti-obesity effects [33]. In our study, the *L. reuteri* MBHC 10138 strain, which had been isolated from breast milk, demonstrated its ability to reduce lipid accumulation in vitro, raising its possible contribution to weight regulation in infants by influencing the lipid metabolism. This action may depend on the strain’s viability, necessitating formulation techniques such as freeze-drying to ensure its preservation. Further research is needed to explore this potential link and understand the mechanisms by which MBHC 10138 could help prevent obesity in early childhood.

## 4. Materials and Methods

### 4.1. Strains and Reagents

Strains MBHC 10138 and MJ-1 were provided by Microbiohealthcare Co., Ltd., from a microbial collection bank (Yongin, Republic of Korea). The commercial strain was isolated from a commercial probiotic product (Biogaia Protectis Baby Drop, Stockholm, Sweden), identified via 16S rDNA sequencing. Meanwhile, *L. reuteri* KACC 11452 was purchased from the Korean Agricultural Culture Collection (KACC, Wanju-gun, Republic of Korea). All the strains were cultured in De Man–Rogosa–Sharpe (MRS, Difco, Tucker, GA, USA) medium at 37 °C for 24~48 h. For long-term storage, the bacterial cells were suspended in 20% glycerol and stored at −80 °C.

All the chemicals and reagents used in this work were purchased either from Sigma-Aldrich Co. (St. Louis, MO, USA) or Merck (Darmstadt, Germany).

### 4.2. Identification of MBHC 10138 Strain by 16S rDNA Sequencing and Phylogenetic Analysis

To identify the breast milk-isolated MBHC 10138 strain, the 16S rDNA sequence was used as a marker gene. Genomic DNA was isolated using the Wizard DNA extraction kit (Promega, Madison, WI, USA). The 16S rDNA sequence was then amplified using universal primers 27F (5′-AGAGTTTGATCCTGGCTCAG-3′) and 1492R (5′-TACGGYTACCTTGTTACGACTT-3′) and subsequently sequenced by Biofact Co. Ltd. (Daejeon, Republic of Korea). The resulting sequence was analyzed using the EzBioCloud database (ChunLab Inc., Seoul, Republic of Korea) for species identification [15].

For the phylogenetic analysis of MBHC 10138, the 16S rDNA sequences of closely related strains were aligned. A phylogenetic tree was constructed using the neighbor-joining method in the Molecular Evolutionary genetics analysis (MEGA) software (Version 11). The robustness of individual branches was evaluated by the bootstrapping of 1000 replications.

### 4.3. Safety Assessment and Probiotic Characterization

#### 4.3.1. Biogenic Amine Production Analysis

For the evaluation of biogenic amine production, the MBHC 10138 strain was cultured on decarboxylase medium agar, supplemented with various amino acids as previously described [34]. The decarboxylase medium agar was prepared using a formula containing tryptone (0.5%), yeast extract (0.5%), meat extract (0.5%), sodium chloride (0.25%), glucose (0.05%), tween 80 (0.1%), magnesium sulfate (0.02%), manganese sulfate (0.005%), ferrous sulfate (0.004%), ammonium citrate (0.2%), thiamine (0.001%), dipotassium phosphate (0.2%), calcium carbonate (0.01%), pyridoxal 5-phosphate (0.005%), bromocresol purple (0.006%), agar (2%), and a pH of 5.3. Different amino acids (L-tyrosine, L-histidine, L-ornithine, L-phenylalanine, and L-lysine) were added to the decarboxylase medium at a ratio of 1% [35], while a medium without amino acids was used as a control. *L. reuteri* MBHC 10138 was streaked onto the plates and incubated aerobically at 37 °C for 48 h. Biogenic amine production was assessed based on the appearance of a purple color around the colonies.

#### 4.3.2. Hemolytic Activity Analysis

To evaluate the strain’s hemolytic activity, *L. reuteri* MBHC 10138 was streaked on blood agar (Hardy Diagnostics, Santa Maria, CA, USA, A10) with 5% defibrinated sheep blood and incubated at 37 °C for 24 h. Its hemolytic activity was assessed under transmitted light, identified by the appearance of clear zones around the colonies, indicating the breakdown of red blood cells. *Escherichia coli* O138 was used as a control under the same conditions.

#### 4.3.3. D-Lactic Acid Production Test

To evaluate D-lactate production, the *L. reuteri* MBHC 10138 strain was cultured in MRS medium at 37 °C for 48 h. The supernatant was then analyzed using a D-lactic acid assay kit (Megazyme, K-DATE, Wicklow, Ireland), according to the manufacturer’s instructions [33].

#### 4.3.4. Bile Salt Deconjugation Test

Bile salt deconjugation was assessed using a modified standard plate assay method [36]. *L. reuteri* MBHC 10138 strains were inoculated onto MRS agar plates supplemented with 0.5% taurodeoxycholic acid sodium salt hydrate (Sigma-Aldrich, Saint Louis, MO, USA), incubated at 37 °C for 48 h. Deconjugation activity was indicated by the presence of a visible halo and opaque white precipitate around the colonies.

#### 4.3.5. Antibiotic Susceptibility Test

The antibiotic susceptibility of the candidate strains was determined by measuring the minimum inhibitory concentration (MIC) using the two-fold broth dilution method [33,37]. The antibiotics tested included chloramphenicol, ampicillin, tetracycline, gentamycin, kanamycin, streptomycin, erythromycin, and clindamycin, in concentrations ranging from 0.25 to 256 μg/mL, following the guidelines of the European Food Safety Authority (EFSA) [33]. The EFSA-recommended MIC cut-off values for *L. reuteri* were used to assess the antibiotic susceptibility of MBHC 10138.

#### 4.3.6. Oral–Gastric–Intestinal Transit Assay

An oral–gastric–intestinal (OGI) transit assay was conducted with modifications based on a previously described method [38]. Initially, the *L. reuteri* strains were exposed to oral stress by treating the bacterial cells (10^9^ CFU/mL) in an oral stress solution (NaCl, 6.2 g/L; KCl, 2.2 g/L; CaCl_2_, 0.22 g/L; NaHCO_3_, 1.2 g/L; and lysozyme, 0.15 g/L) at 37 °C for 10 min. Afterwards, the solution was removed by centrifugation at 1800× *g* for 5 min. The cells were then resuspended in a gastric stress solution with a pH of 3 (NaCl, 6.2 g/L; KCl, 2.2 g/L; CaCl_2_, 0.22 g/L; NaHCO_3_, 1.2 g/L; and pepsin 3 g/L, pH 2), incubated at 37 °C for 30 min, followed by treatment in a gastric stress solution with a pH of 2 for 30 min. After removing the gastric solution by centrifugation, the cells were exposed to an intestinal stress solution (NaCl, 5 g/L; KCl, 0.6 g/L; CaCl_2_, 0.25 g/L; pancreatin, 1 g/L; bile oxgall, 3 g/L; and pH 7), incubated at 37 °C for 120 min. The cell viability at each step was determined by plating the cells on an MRS medium using the serial dilution method, counting the number of colony-forming units (CFUs) after 48 h.

### 4.4. Purine Degradation by L. reuteri MBHC 10138 and Related Strains

#### 4.4.1. Purine Degradation Assay

Purine degradation was assessed as described before, with some modifications [39]. In brief, guanosine and inosine solutions were prepared by dissolving 1.25 mM of guanosine and inosine in 100 mM of potassium phosphate buffer (pH 7.0). Adenosine solution was prepared by dissolving 1.25 mM of adenosine in the phosphate buffer. The *L. reuteri* strains were cultured in MRS medium and incubated at 37 °C for 24 h. After incubation, the cells were harvested by centrifugation, washed twice with PBS, and suspended at a cell density of 10^9^ cfu/mL in different nucleoside solutions. After incubation at 37 °C for 30 min, the bacterial suspension was centrifuged at 4000× *g* for 10 min. Then, the supernatant (270 μL) was taken and mixed with 30 μL of 0.1 M HClO_4_ solution to stop the reaction. The remaining nucleoside in the supernatant was determined by HPLC.

To further differentiate the degradation capabilities of the various strains, the cell density was decreased to 10^8^ cfu/mL and 10^7^ cfu/mL for the degradation of guanosine/inosine and adenosine, respectively, and the incubation time was decreased to 15 min.

#### 4.4.2. HPLC Analysis

The concentration in the supernatant was determined using an HPLC system equipped with a binary HPLC pump (Waters 1525, Milford, MA, USA) connected to a YMC ODS column (S-5 μm, 12 mm, 250 × 4.6 mm). Isocratic elution was performed with 20 mM of phosphate buffer mixed with 4% methanol, and the flow rate was set at 1 mL/min. Guanosine, inosine, and adenosine were detected at 254 nm and quantified via comparison with the standard compounds.

### 4.5. Analysis of Inhibitory Activity against Streptococcus Mutans KCTC3065 Biofilm

Biofilm inhibition was determined as previously described [40]. *L. reuteri* MBHC 10138 was cultured in MRS medium, while *S. mutans* KCTC 3065 was cultured in BHI medium at 37 °C for 24 h. They were then adjusted to an OD_600_ of approximately 1 and diluted 100 fold using a BHI medium supplemented with 0.2% sucrose. A biofilm assay was carried out in 96-well plates; each well contained 160 μL of BHI broth (supplemented with 0.2% sucrose) and was then inoculated with 20 μL of *L. reuteri* MBHC 10138 and 20 μL of *S. mutans* diluent. The plates were incubated at 37 °C for 24 h under anaerobic conditions in a GasPak system containing an anaerobic gas-generating pouch (BD, Mississauga, ON, Canada).

After incubation, the supernatants were discarded, and the well was washed twice with PBS. The biofilm at the bottom of the well was stained with 125 μL of 0.1% safranin for 30 min. After washing with distilled water, the dye was extracted with 125 μL of 33% acetic acid. The biofilm biomass was measured at 492 nm absorbance with a microtiter plate reader (Tecan, Manne Dorf, Switzerland).

### 4.6. Assay of L. reuteri Strains on Lipid Accumulation in HepG2 Cells

#### 4.6.1. Cell Culture

HepG2 cells, a human hepatocellular liver carcinoma cell line, were purchased from the Korean Cell Line Bank (Seoul, Republic of Korea). Lipid accumulation was induced as in a previous study [33,38]. Briefly, the cells were cultured in Dulbecco’s Modified Eagle Medium (DMEM) containing 10% fetal bovine serum (FBS) and 1% penicillin–streptomycin (10,000 U/mL), purchased from Gibco (Avenue, Waltham, MA, USA), in an incubator at 37 °C under a humidified air atmosphere containing 5% CO_2_.

#### 4.6.2. Sample Treatment

HepG2 cells were seeded at a density of 5 × 10^5^ cells/mL in six-well plates, incubated for 24 h, starved overnight in FBS-free DMEM, and washed three times with PBS before treatment with the *L. reuteri* strains. The MBHC 10138, MJ-1, KACC 11452, and commercial strains were individually suspended in fresh antibiotic-free DMEM, supplemented with a fatty solution (1 mM oleic acid, 7.5 μg/mL cholesterol, 1% BSA) at a concentration of 10^9^ CFU/mL. The bacterial suspensions were transferred to a transwell membrane (SPL, Pochon, Republic of Korea) in the six-well culture plate containing the HepG2 cells and incubated together for 6 h. The cells treated solely with the fatty solution were used as a negative control, while those receiving simvastatin (1 μM) were positive controls. After 6 h of co-culture, the transwell membranes were removed, and the HepG2 cells were washed and stained.

#### 4.6.3. Staining and Quantification of Lipid Accumulation

Staining of the lipid droplets accumulated in the HepG2 cells was carried out as described by a previous work [41]. Briefly, the cells were washed with phosphate-buffered saline (PBS) and fixed with 4% formaldehyde for 20 min. After the removal of the formaldehyde, the cells were washed with 60% isopropanol for 5 min and then dyed with an Oil Red O (ORO) working solution, obtained from a stock solution prepared by dissolving 0.5 g ORO powder in isopropanol and filtering the product. Then, the ORO working solution was freshly concocted by mixing the stock solution with distilled water in a ratio of 3:2, followed by filtering. After 30 min, the dye was removed, and the cells were washed with 60% isopropanol. The dye retained in the cells was extracted by adding 100% isopropanol, shaking for 5 min, and then quantified at 510 nm with a microplate reader (Tecan, Spectrofluor Plus, Maennedorf, Switzerland).

## 5. Conclusions

In conclusion, *L. reuteri* MBHC 10138, a strain isolated from human breast milk, exhibited key characteristics of a safe and effective probiotic with multiple beneficial biological functions. This strain demonstrated little D-lactate and biogenic amine production, bile salt deconjugation, and hemolytic activity, indicating its safety profile. Additionally, it was susceptible to most antibiotics and capable of surviving under simulated oral–gastric–intestinal conditions. More importantly, *L. reuteri* MBHC 10138 displayed better biological activities than the reference strains in terms of purine assimilation and the inhibition of biofilm formation, and lipid accumulation. These results suggest this strain’s potential as a candidate for the development of functional probiotics for managing hyperuricemia, oral caries, and obesity. Further studies are needed to explore the in vivo efficacy and mechanisms of *L. reuteri* MBHC 10138 function in depth.

## Figures and Tables

**Table 1:** Safety assessment of *L. reuteri* MBHC 10138.

Safety Test	*L. reuteri* MBHC 10138	*L. rhamnosus* GG
Hemolytic activity	-	-
D-lactate production (g/L)	18.39	23.11
Bile salt deconjugation	-	-
Bioamine production		
L-Histidine	-	-
L-Tyrosine	-	-
L-Phenylalanine	-	-
Arginine	-	-
Tryptophan	-	-
L-Ornithine	-	-
Antibiotic susceptibility to		
Ampicillin	32 (R)	1
Clindamycin	0.25	1
Chloramphenicol	4	4
Erythromycin	1	1
Gentamycin	256 (R)	32 (R)
Kanamycin	>256 (R)	R
Streptomycin	128 (R)	32 (R)
Tetracycline	16	1

-, no activity; R, resistant to the antibiotic based on the MIC value (μg/mL) recommended by the European Food Safety Authority (EFSA) in 2012.

**Table 2:** *L. reuteri* MBHC 10138’s viability after oral–gastric–intestinal transit assay.

OGI Transit (Log10 CFU/mL)	*L. reuteri* MBHC 10138	*L. rhamnosus* GG
Initial	9.16 ± 0.021	9.21 ± 0.023
Oral stress	9.14 ± 0.048	9.21 ± 0.006
Gastric stress (pH3)	9.11 ± 0.04	9.17 ± 0.103
Gastric stress (pH2)	9.04 ± 0.030	9.01 ± 0.035 *
Intestinal stress	8.58 ± 0.050 ****	7.98 ± 0.045 ****

* *p* < 0.05, and **** *p* < 0.0001, compared to the initial value.

## Data Availability

Data are contained within the article and Supplementary Materials.

## Supplementary Materials

The following supporting information can be downloaded at https://www.mdpi.com/article/10.3390/antibiotics13100964/s1, Figure S1. Assessment of biogenic amine production of *Limosilactobacillus reuteri* MBHC 10138 on the decarboxylase medium. Medium without any amino acid served as control (A). *L. reuteri* was streaked on the control and medium supplemented with L-arginine (B), L- histidine (C), L-lysine (D), L-ornithine (E), L-phenylalanine (F), L-tyrosine (G), L-tryptophan (H). The plates were incubated at 37 °C for 48 h. Figure S2. Assessment of hemolytic activity of *Limosilactobacillus reuteri* MBHC 10138 on the blood agar medium. *Limosilactobacillus reuteri* MBHC 10138 showed no hemolytic activity (A). In contrast, *E. coli* O138 served as a positive control, showing a halo zone around the colony due to the β-hemolysis (B). Figure S3. The morphology of *Limosilactobacillus reuteri* MBHC 10138 (A), *L. reuteri* commercial strain (B), *L. reuteri* KACC 11452 (C) and *L. reuteri* MJ-1 (D) in MRS agar plates. The plates were incubated at 37 °C for 48 h.
