# Supplementary material for: Characterization of Human Breast Milk-Derived Limosilactobacillus reuteri MBHC 10138 with Respect to Purine Degradation, Anti-Biofilm, and Anti-Lipid Accumulation Activities
Source: Antibiotics (Basel). 2024 Oct 12;13(10):964. doi: 10.3390/antibiotics13100964 (PMC11504937; doi:10.3390/antibiotics13100964)
Supplement: Supplementary file 1 [file antibiotics-13-00964-s001.zip › antibiotics-3218921-supplementary.pdf]

Supplementary data

Figure S1

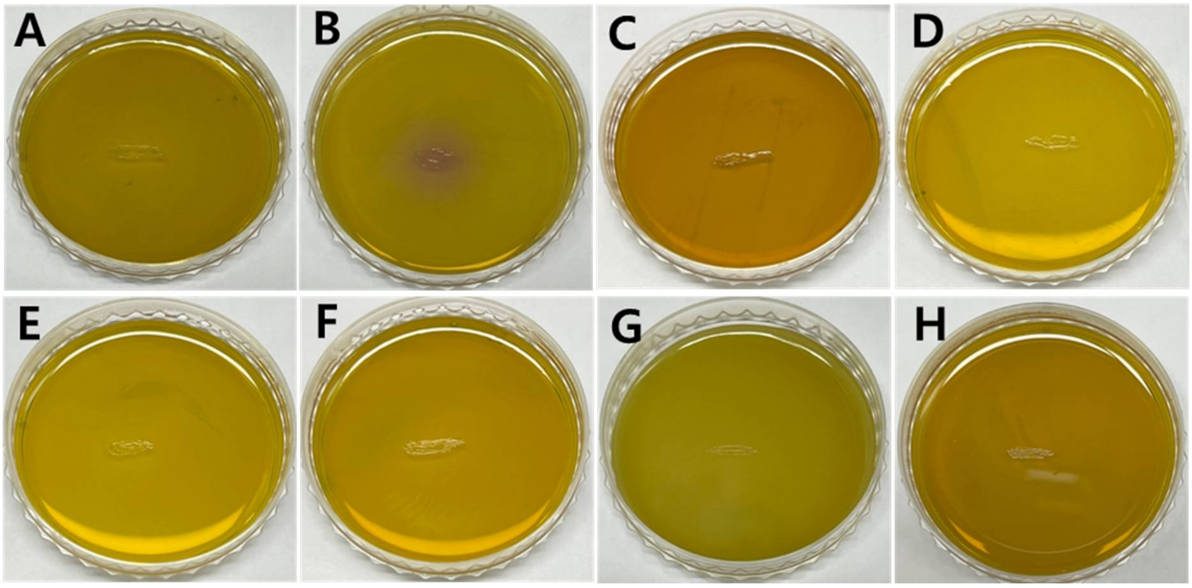

Figure S1. Assessment of biogenic amine production of *Limosilactobacillus reuteri* MBHC 10138 on the decarboxylase medium. Medium without any amino acid served as control (A). *L. reuteri* was streaked on the control and medium supplemented with L-arginine (B), L- histidine (C), L-lysine (D), L-ornithine (E), L- phenylalanine (F), L-tyrosine (G), L-tryptophan (H). The plates were incubated at 37°C for 48 h.

Figure S2.

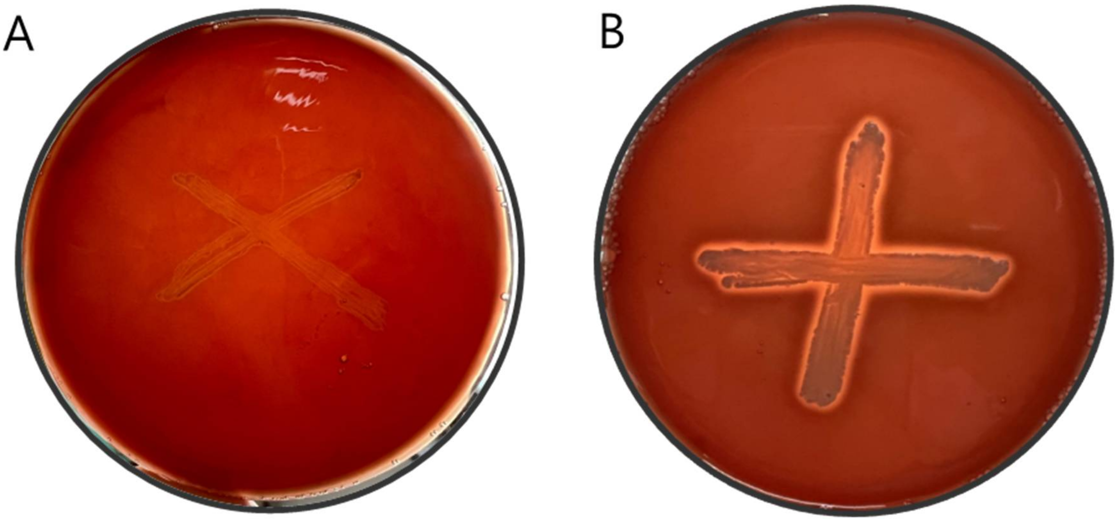

Figure S2. Assessment of hemolytic activity of *Limosilactobacillus reuteri* MBHC 10138 on the blood agar medium. *Limosilactobacillus reuteri* MBHC 10138 showed no hemolytic activity (A). In contrast, *E. coli* O138 served as a positive control, showing a halo zone around the colony due to the β-hemolysis (B).

Figure S3

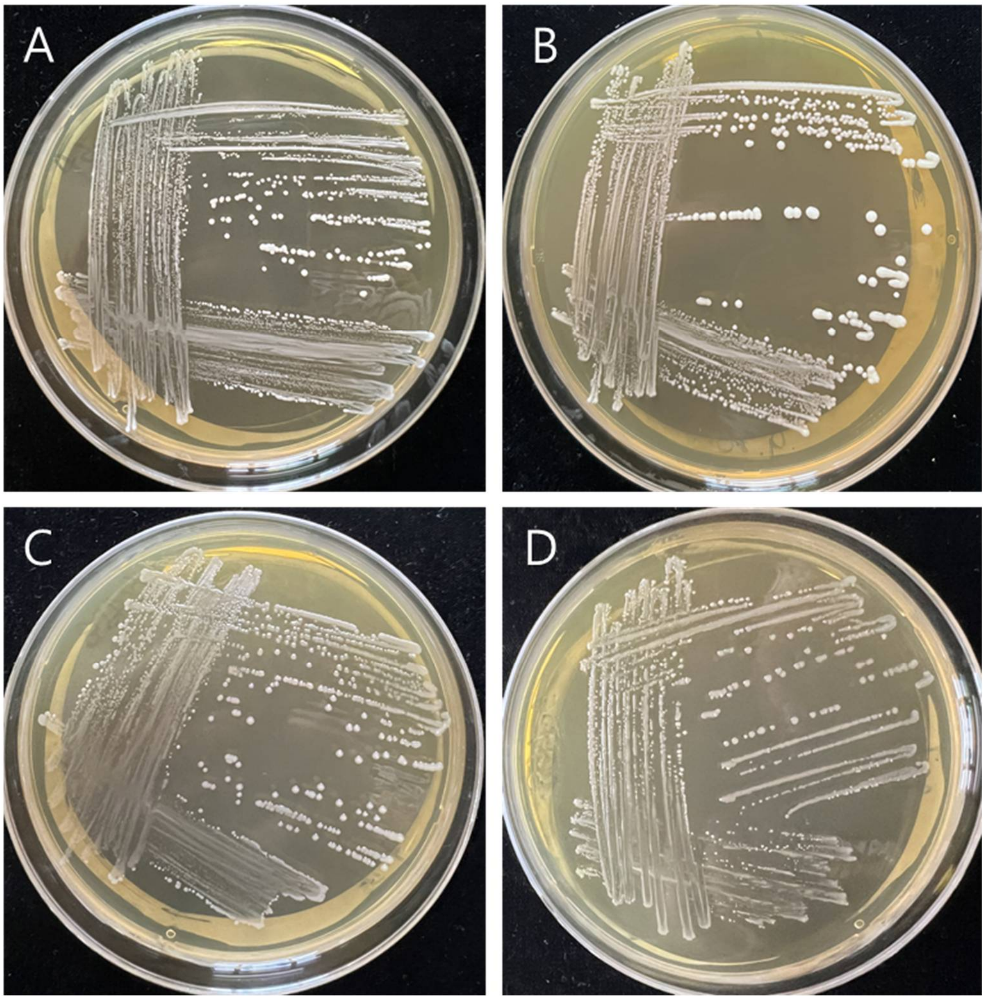

Figure S3. The morphology of *Limosilactobacillus reuteri* MBHC 10138 (A), *L. reuteri* commercial strain (B), *L. reuteri* KACC 11452 (C) and *L. reuteri* MJ-1 (D) in MRS agar plates. The plates were incubated at 37°C for 48 h.
